# Polygenic power calculator: Statistical power and polygenic prediction accuracy of genome-wide association studies of complex traits

**DOI:** 10.3389/fgene.2022.989639

**Published:** 2022-10-10

**Authors:** Tian Wu, Zipeng Liu, Timothy Shin Heng Mak, Pak Chung Sham

**Affiliations:** ^1^ Department of Psychiatry, Li Ka Shing Faculty of Medicine, The University of Hong Kong, Pok Fu Lam, Hong Kong SAR, China; ^2^ State Key Laboratory of Brain and Cognitive Sciences, The University of Hong Kong, Pok Fu Lam, Hong Kong SAR, China; ^3^ Centre for PanorOmic Sciences, Li Ka Shing Faculty of Medicine, The University of Hong Kong, Pok Fu Lam, Hong Kong SAR, China; ^4^ Fano Labs, Hong Kong, Hong Kong SAR, China

**Keywords:** GWAS, polygenic model, power calculation, online tool, statistical method

## Abstract

Power calculation is a necessary step when planning genome-wide association studies (GWAS) to ensure meaningful findings. Statistical power of GWAS depends on the genetic architecture of phenotype, sample size, and study design. While several computer programs have been developed to perform power calculation for single SNP association testing, it might be more appropriate for GWAS power calculation to address the probability of detecting any number of associated SNPs. In this paper, we derive the statistical power distribution across causal SNPs under the assumption of a point-normal effect size distribution. We demonstrate how key outcome indices of GWAS are related to the genetic architecture (heritability and polygenicity) of the phenotype through the power distribution. We also provide a fast, flexible and interactive power calculation tool which generates predictions for key GWAS outcomes including the number of independent significant SNPs, the phenotypic variance explained by these SNPs, and the predictive accuracy of resulting polygenic scores. These results could also be used to explore the future behaviour of GWAS as sample sizes increase further. Moreover, we present results from simulation studies to validate our derivation and evaluate the agreement between our predictions and reported GWAS results.

## Introduction

Genome-wide association studies (GWAS) aim to systematically identify single-nucleotide polymorphisms (SNPs) associated with complex phenotypes. Though not necessarily causal, associated SNPs are good starting points for elucidating biological mechanisms of diseases and related phenotypes. GWAS on a wide range of phenotypes have confirmed the polygenic nature of most common traits, with thousands of SNPs each making a small contribution to individual differences in the population (Visscher et al., 2017). The recent increase in the sample size of GWAS and meta-GWAS has resulted in more of these SNPs to be identified, leading not only to more comprehensive understanding of disease etiology (Cano-Gamez and Trynka, 2020), but also greater accuracy in the calculation of polygenic scores to predict individual genetic liability to develop disease (Vilhjalmsson et al., 2015; Mak et al., 2017; Torkamani et al., 2018).

Adequate statistical power is necessary to both detect enough SNPs to inform etiology and to obtain accurate effect size estimate for polygenic score calculations (Dudbridge, 2013). Several computer programs have been developed to perform power calculation for single SNP association testing. For example, Genetic Power Calculator (GPC) (Purcell et al., 2003) used closed-form analytic results (Sham and Purcell, 2014) to perform power calculations for linkage and association studies. Genetic Association Study Power Calculator (GAS) (Johnson and Abecasis, 2017) performs power calculation for genetic association studies under case-control design. However, these tools perform power calculation for single SNPs, ignoring the polygenic nature of complex diseases, and the simultaneous testing of millions of SNPs that is now standard in GWAS (Sham and Purcell, 2014). Meta-GWAS Accuracy and Power (MetaGAP) (de Vlaming et al., 2017) performs GWAS power calculations and introduces genetic correlation parameters to account for effect size heterogeneity between studies. However, it is restricted to quantitative phenotype and random samples.

Since the goal of GWAS is to detect any truly associated SNPs, power calculation might more appropriately address the probability of detecting any number of associated SNPs, than the probability of detecting a specific associated SNP. Such a calculation would require specification of the entire distribution of effect size of all analysed SNPs, rather than the effect size of a single SNP. Several methods have been proposed to infer the underlying genetic effect size distribution based on significant GWAS hits or GWAS summary statistics (Park et al., 2010; So et al., 2010; Chatterjee et al., 2013; Moser et al., 2015; Zhang et al., 2018). Evidence shows that a point-normal distribution is adequate to fit the distribution of true effects of common variants for some complex traits (Zhang et al., 2018) and it is more practical than the infinitesimal model (Visscher et al., 2017).

This report describes a fast, flexible and interactive power calculation tool for GWAS under the assumption of a point-normal distribution of standardized effect sizes. The program generates predictions for the key outcomes of GWAS, including the distribution of statistical power across all independent causal SNPs, the expected number of independent genome-wide significant SNPs, total phenotypic variance explained by these SNPs, and the predictive accuracy of optimally weighted polygenic scores (PGS). It allows the user to specify the nature of the phenotype under consideration (quantitative or dichotomous), its epidemiological features (e.g., disease prevalence) and genetic architecture (e.g., SNP-heritability), and the study design (e.g., case-control).

## Material and methods

The input parameters and the output indices of the program are summarized in Table 1.

**TABLE 1:** Key input parameters and output indices.

General parameters	
*n*	GWAS sample size
*m*	Number of nearly independent SNPs, after removing SNPs in strong LD
*h* ^*2*^	SNP heritability of quantitative phenotype or of liability to disease
*π*_*0*_	Proportion of SNPs that do not contribute to SNP heritability
Parameter in qualitative phenotype model	
*K*	Population disease prevalence
Study design parameters	
$T_{L}$	Lower threshold for extreme sample selection
$T_{U}$	Upper threshold for extreme sample selection
$P_{L}$	Proportion of samples below $T_{L}$ , in extreme sample selection
ω	Proportion of cases in case-control design
Output indices	
E(S)	Expected number of independent significant SNPs
E(C)	Expected number of detected causal SNPs
$\underset{j\in \Omega }{\sum }{\hat{\beta }}_{j}^{2}$	Apparent phenotypic variance explained by independent significant SNPs
$\underset{j\in \Omega }{\sum }{\left[E\left(\beta_{j}^{2}|{\hat{\beta }}_{j}\right)\right]}^{2}$	Corrected phenotypic variance explained by the independent significant SNPs

### Model description

The phenotype is either an observed quantitative trait or a disease determined by a latent continuous liability (Falconer, 1965). For simplicity, SNPs are assumed to have been made nearly independent by clumping or pruning; the total number of SNPs (*m*) is the effective number of independent SNPs in the entire genome. A proportion π _0_ of independent SNPs do not contribute to phenotypic variance (i.e., the null SNPs), while the remaining $\left(1-\pi_{0}\right)\times m$ SNPs are causally associated with the phenotype (i.e., the non-null SNPs), explaining a proportion $h^{2}$ of the phenotypic variance, known as the SNP heritability. The effect size of a SNP *j* on phenotype (observed or latent), $\beta_{j}$ , is defined as the regression coefficient of the standardized quantitative phenotype on the standardized genotype. The effect sizes of causal SNPs are assumed to be drawn from a normal distribution with mean zero and variance $\frac{h^{2}}{m\left(1-\pi_{0}\right)}$ . Overall, the distribution of effect sizes of all SNPs follow a point-normal distribution:

$$\beta \;∼\;\pi_{0}\delta_{0}+\left(1-\pi_{0}\right)N\left(0,\frac{h^{2}}{m\left(1-\pi_{0}\right)}\right)$$

where $\delta_{0}$ denotes a point mass at zero. When π _0_ is zero, effect sizes become normally distributed, corresponding to the infinitesimal model (Falconer, 1996).

For disease phenotypes, standardised log-odds ratios ( $\gamma_{j}$ ) from the logistic regression model can be transformed approximately to effect size on the liability scale ( $\beta_{j}$ ), assuming knowledge of disease prevalence *K* in the population (Wu and Sham, 2021).

$$\beta_{j}\approx \frac{K\left(1-K\right)}{\phi \left(\Phi^{-1}\left(K\right)\right)}\gamma_{j}$$

where ϕ is the standard normal probability density function and $\Phi^{-1}$ is the inverse of the standard normal cumulative distribution function.

### Distribution of effect size estimates

**FIGURE 1:**
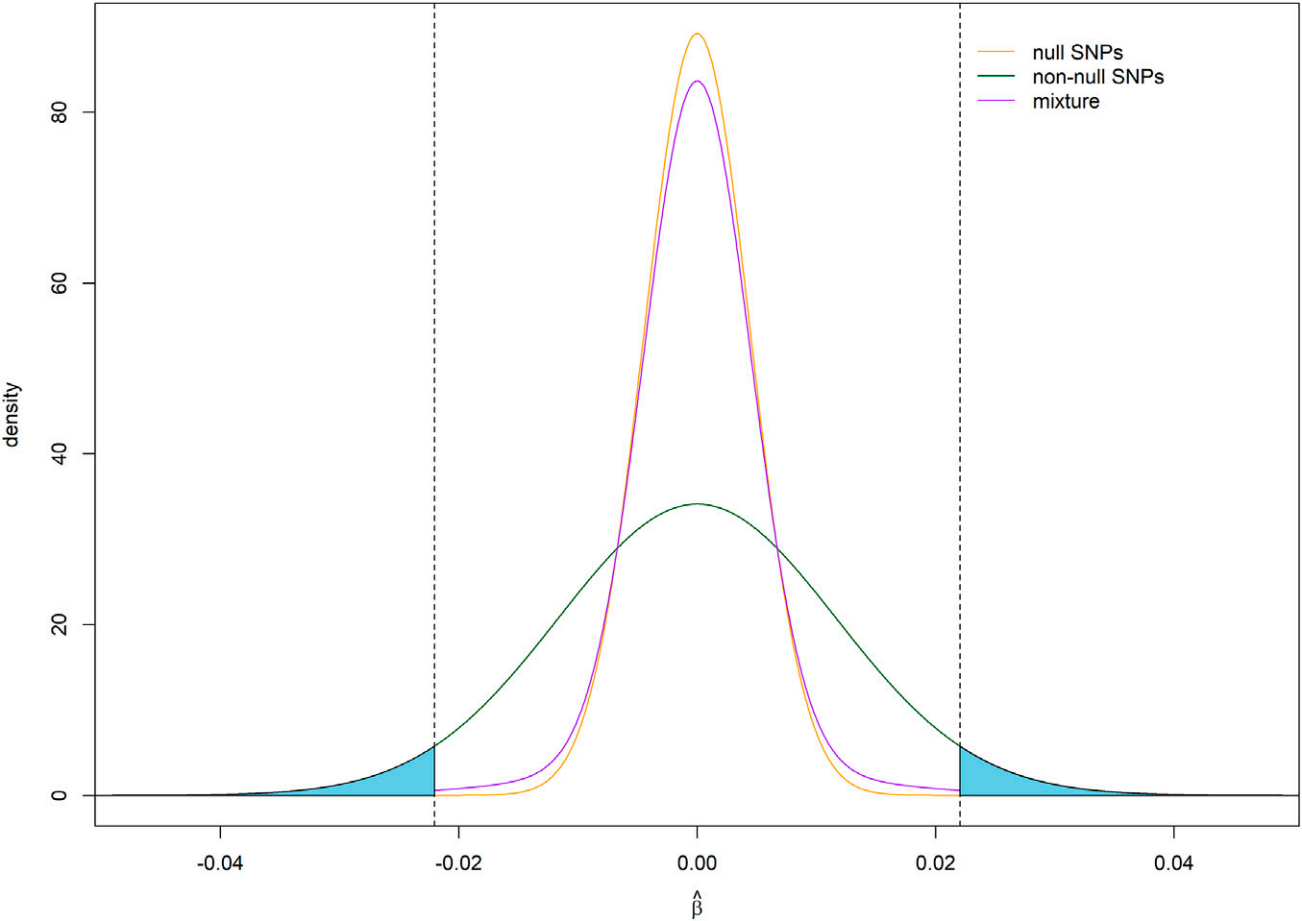
Assumed distribution of effect size estimates under a point-normal model. For illustration, the critical values for statistical significance are shown as vertical dotted lines, while average statistical power for detecting non-null SNPs is given by the shaded areas under density curve for non-null SNPs. Parameter values $h^{2}$ = 0.7, *m* = 60,000, $\pi_{0}$ = 0.9, *n* = 50,000, α = 5 × 10^−8^.

For quantitative traits, the regression coefficient estimate ${\hat{\beta }}_{j}$ for a SNP with a true effect size $\beta_{j}$ is normally distributed with mean $\beta_{j}$ and variance approximately $\frac{1}{n}$ , where *n* is the sample size (Dudbridge, 2013). Thus, the overall distribution of $\hat{\beta }$ is a mixture of two normal distributions (Figure 1):

$$\hat{\beta }\;∼\;\pi_{0}\;N\left(0,\frac{1}{n}\right)+\left(1-\pi_{0}\right)\;N\left(0,\frac{h^{2}}{m\left(1-\pi_{0}\right)}+\frac{1}{n}\right)$$

For binary traits, the sampling variance of the per-standard deviation effect estimate on the liability scale depends on the disease prevalence (*K*) in the population and the proportion of cases ( w ) in the sample, as well as the total (case and control) sample size, as follows (Wu and Sham, 2021):

$$Var\left(\hat{\beta }\right)\approx Var\left(\beta \right)+\frac{1}{n}\frac{K^{2}{\left(1-K\right)}^{2}}{w\left(1-w\right)}\frac{1}{\phi^{2}\left(\Phi^{-1}\left(K\right)\right)}$$

The sample size *n* can be rescaled by a factor $\frac{w\left(1-w\right)}{K^{2}{\left(1-K\right)}^{2}}\phi^{2}\left(\Phi^{-1}\left(K\right)\right)$ to obtain the size of a random sample with equivalent sampling variance for $\hat{\beta }$ , for an observed quantitative trait with the same parameters ( π _0_ ,m, and $h^{2}$ ) as the disease liability.

### Distribution of statistical power across causal single-nucleotide polymorphisms

The statistical power for an individual SNP is determined by its effect size, the sample size, and the desired significance level. In a random sample of size n , the test statistic for the association between a quantitative phenotype and a SNP is $\hat{\beta }\sqrt{n}$ , which approximately follows a non-central chi-squared distribution with non-centrality parameter (NCP) $n\beta^{2}$ . The statistical power of detecting a SNP is given by the tail area of this distribution beyond the critical value for the desired significance level. Thus, given an assumed distribution of β across all non-null SNPs, we can obtain the distribution of statistical power, for any sample size and desired level of statistical significance. This was done by partitioning possible β values, for example, [−10 sd,10 sd] of the assumed effect size distribution, into narrow intervals, and calculating the probability of the effect size to be within intervals and the statistical power for an effect size at the mid-point of the intervals. This method provides increasingly more accurate approximations to the probability density function of statistical power as the intervals become narrower. Based on this approximate probability density function of statistical power, we calculated the average and variance of statistical power across causal SNPs ( E(p) and Var(p) ).

### Distribution of the number of and variance explained by independent significant single-nucleotide polymorphisms

From the expectation and variance of statistical power, we derived formulae for the expectation and variance of the number of independent significant SNPs, as well as the proportion of phenotypic variance explained by these SNPs. These formulae were validated by simulation studies. For SNP j, (j=1,2,…m) , we generated minor allele frequency $f_{j}∼Uniform\;\left(0.01,0.5\right)$ and independent genotype value $X_{j}∼Binomial\;\left(2,f_{j}\right)$ (subsequently standardised to have mean zero and variance one). We randomly selected $m\left(1-\pi_{0}\right)$ SNPs to be causal, with standardised effect size $\beta_{j}∼$ $N\left(0,\frac{h^{2}}{m\left(1-\pi_{0}\right)}\right)$ ; the remaining $m\pi_{0}$ SNPs were assigned effect size zero. We also generated error term $\varepsilon ∼N\left(0,1-h^{2}\right)$ , which was added to the total effect of the causal SNPs to calculate the phenotypic value of each individual. We then performed association analysis for SNPs to obtain the estimated effect sizes ${\hat{\beta }}_{j}$ and associated *p*-values. This procedure was repeated 100 times using LDAK (Speed et al., 2017), and the results were checked for consistency with the theoretical number of significant SNPs and its 95% probability interval calculated by our formulae.

### Polygenic score predictive accuracy

The polygenic model specifies that the phenotypic value is related to SNP genotypes by $y_{i}=G_{i}+\varepsilon_{i}$ , where $G_{i}=\sum_{j=1}^{m}{\beta_{j}x}_{ij}$ is defined as the true additive genetic value of individual *i*, and *m* is the number of SNPs. In practice, the true effect size $\beta_{j}$ are unknown, and we calculate individual PGS using estimates of $\beta_{j}$ as weights, i.e., ${\tilde{G}}_{i}=\sum_{j=1}^{m}{{\tilde{\beta }}_{j}x}_{ij}$ .

A number of different methods to determine the weights ${\tilde{\beta }}_{j}$ have been proposed. The simplest method is to use the regression coefficient estimates ( ${\hat{\beta }}_{j}$ ) from simple linear or logistic regression of the phenotype, on each SNP separately. When the SNPs are independent and both phenotype and genotype data are standardised to have mean 0 and variance 1, the sampling variance of the regression coefficient estimate for a quantitative phenotype is $Var\left({\hat{\beta }}_{j}\right)=\frac{\sigma_{e}^{2}}{\overset{n}{\underset{i=1}{\sum }}{\left(x_{ij}-\bar{x}\right)}^{2}}=\frac{\sigma_{e}^{2}}{\left(n-1\right)s^{2}}\approx \frac{\sigma_{e}^{2}}{n}\approx \frac{1}{n}$ and the efficacy of PGS relative to the true additive genetic value is $r^{2}\left({\hat{G}}_{i},G_{i}\right)=\frac{1}{1+\frac{m}{nh^{2}}},\;i=1,2,\ldots n$ (Daetwyler et al., 2008), where ${\hat{G}}_{i}$ denotes the PGS constructed by ${\hat{\beta }}_{j}$ . The prediction accuracy of PGS on phenotype, i.e., $r^{2}\left({\hat{G}}_{i},y_{i}\right),$ is then given by $r^{2}\left({\hat{G}}_{i},G_{i}\right)h^{2}$ (Wray et al., 2013). Furthermore, the prediction accuracy of PGS for binary phenotypes on the liability scale can be easily obtained based on the aforementioned effect size transformation. Once the variance explained on the liability scale is obtained, it can be easily transformed to the area under the curve (AUC) of receiver-operator characteristic (ROC) or Nagelgerke’s pseudo- $R^{2}$ following Lee et al. (2012). However, the marginal effect estimates are poor proxies of true SNP effect sizes. Also, not all SNPs contribute to the phenotypic variance, so only a number of SNPs should be included in the PGS. To address these issues, shrinkage methods to construct PGS have been proposed (Purcell et al., 2009; Vilhjalmsson et al., 2015; Bigdeli et al., 2016; Mak et al., 2016; So and Sham, 2017; Qian et al., 2020; Song et al., 2020). A classic way of selecting SNPs contributing to PGS is *p*-value thresholding (Euesden et al., 2015), where only SNPs with GWAS *p*-value less than a certain threshold are retained, in effect shrinking the regression coefficient estimates of SNPs with *p*-value above the threshold to zero. The threshold is usually determined by optimizing the PGS prediction accuracy of the target phenotype by split-sample or out-sample validation. Another, more sophisticated, shrinkage method is to replace the regression coefficient by the posterior expectation $E\left(\beta_{j}|{\hat{\beta }}_{j}\right)$ , assuming a certain prior distribution for $\beta_{j}$ (Vilhjalmsson et al., 2015; Lloyd-Jones et al., 2019; Song et al., 2020). Thus the magnitude of shrinkage depends on the value of ${\hat{\beta }}_{j}$ non-linearly, with small values being shrunk to zero while large values are relatively unchanged. The efficacy of PGS constructed by various shrinkage methods can be calculated by $r^{2}\left(G_{i},{\tilde{G}}_{i}\right)=\frac{{Cov}^{2}\left(\beta_{j},{\tilde{\beta }}_{j}\;\right)}{Var\left(\beta_{j}\right)Var\left({\tilde{\beta }}_{j}\;\right)}$ , where ${\tilde{G}}_{i}$ denotes the estimated PGS constructed by shrunk estimators of ${\hat{\beta }}_{j}.$ Numeric method is adopted to calculate this efficacy index given the parameters in the genetic effect-size distribution.

### Other study designs and meta-genome wide association studies analysis

We enabled the above framework to be used for power calculation in other study designs, including phenotypic selection of continuous traits (e.g., extreme phenotype design), and case-control studies of binary traits, by deriving the equivalent sample size $n^{*}$ , defined as the sample size that would give the same power to detect associated SNPs as a population study of a continuous phenotype with sample size n . For meta-analysis of case-control studies of a binary trait, we first calculate the equivalent sample sizes of the component studies (which may have different case-control ratios) and then combine them to give a total equivalent sample size.

### Application to real data

We applied our method to four phenotypes including height, body mass index (BMI), major depressive disorder (MDD) and schizophrenia (SCZ) to evaluate how well the predicted GWAS outcomes match up with the reported GWAS outcomes (Wray et al., 2018; Yengo et al., 2018; The Schizophrenia Working Group of the Psychiatric Genomics Consortium Ripke et al., 2020). We selected these four phenotypes because at least three sizeable GWAS or meta-GWAS had been conducted, so that earlier GWAS outcomes could be used to set a reasonable range for π _0_. For example, given Wood et al. (2014) (Wood et al., 2014) reported 623 independent genome-wide significant SNPs detected by meta-analysis for height, we searched for π _0_ such that the 95% probability interval of the predicted number of significant SNPs covered 623. As a result, the range of π _0_ is estimated as [0.6505, 0.6800]. Similarly, we used Locke et al. (2015), Hyde et al. (2016), and Ripke et al. (2014) to estimate the range of π _0_ for BMI, MDD, and SCZ, respectively (Supplementary Table S1).

For SNP heritability, we assumed the latest estimated value reported in literature; when several SNP heritability estimates were reported at about the same time, their average value was used. Specifically, we assumed the SNP heritabilities of height, BMI, MDD, and SCZ were 0.483 (Yengo et al., 2018), 0.249 (see *Web resources*), 0.089 (Howard et al., 2019) and 0.23 (Lam et al., 2019; Lee et al., 2019), respectively. In all of our applications, we set *m* as 60,000 (Wray et al., 2013), assuming meta-analysis samples are from European ancestry. For quantitative trait GWAS using a population cohort, the parameter *n* was simply the sample size of GWAS or meta-GWAS, whereas for binary phenotypes, we used the equivalent sample size described above. If earlier study was a meta-analysis, we calculated the equivalent sample size for each cohort in the meta-analysis, and used the sum of equivalent sample sizes as our model parameter *n* (Supplementary Tables S2, S3). We set the genome-wide significant level *α* as 5 × 10^−8^ except when predicting GWAS key outcomes for height and BMI. For these two studies, *α* was set as 1 × 10^−8^ to be consistent with the literature.

## Results

### Distribution of statistical power across causal single-nucleotide polymorphisms

**FIGURE 2:**
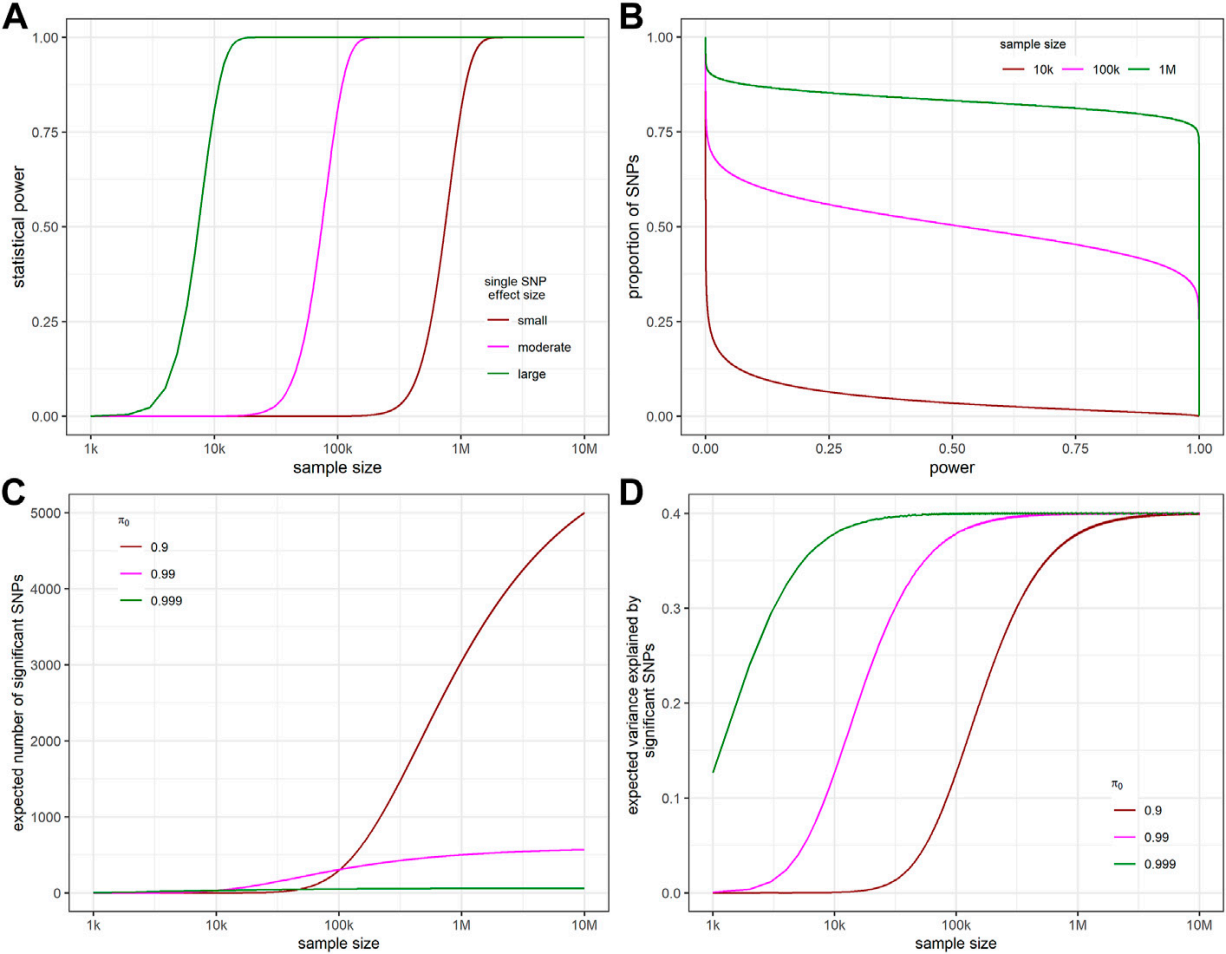
The relationship between statistical power, sample size, expected number of significant SNPs, and apparent variance explained by significant SNPs. **(A)** The relationship between sample size and the statistical power to detect a single SNP with different effect sizes “small”, “moderate”, and “large” representing SNPs that explain 0.01%, 0.1%, and 1% of SNP heritability. **(B)** Proportion of SNPs with at least that level of statistical power on the x-axis for different sample sizes. **(C)** Relationship between expected number of significant SNPs and sample sizes. **(D)** Relationship between the expected variance explained by the significant SNPs and sample sizes. For all figures, $h^{2}$ = 0.4, *m* = 60,000, α = 5 × 10^−8^. For B, $\pi_{0}$ = 0.99.

Our model is based on the assumption that the effect size follows a point-normal distribution. Accordingly, the effect size estimate follows a normal mixture distribution (Figure 1). Figure 2A shows the relationship between statistical power and sample size for different effect sizes for a single SNP. We define SNP explaining 0.01%, 0.1%, and 1% of SNP heritability as having small, moderate and large effect, respectively. When the effect size is large, power curve increased rapidly and saturated soon. The proportion of SNPs with at least that level of statistical power on the x-axis is shown in Figure 2B. This proportion is equivalent to one minus the cumulative probability of power. With the increase of sample size, larger proportions of SNPs remain high statistical power. The expectation and variance of power, given different levels of heritability, $\pi_{0}$ , and sample sizes, are shown in Table 2.

**TABLE 2:** The expectation and variance of statistical power across causal SNPs for different SNP heritability, polygenicity, and sample sizes. *m* = 60,000, α = 5 × 10^−8^.

*h* ^*2*^	*π*_*0*_	Sample size	Expected power	Variance of power
0.1	0.9	10^3^	6.43 × 10^−8^	5.13 × 10^−16^
0.1	0.9	10^5^	8.43 × 10^−4^	7.17 × 10^−5^
0.1	0.9	10^7^	0.67	0.19
0.1	0.99	10^3^	4.49 × 10^−7^	2.96 × 10^−12^
0.1	0.99	10^5^	0.19	0.11
0.1	0.99	10^7^	0.89	0.08
0.1	0.999	10^3^	8.43 × 10^−4^	7.17 × 10^−5^
0.1	0.999	10^5^	0.67	0.19
0.1	0.999	10^7^	0.97	0.03
0.4	0.9	10^3^	1.31 × 10^−7^	3.34 × 10^−14^
0.4	0.9	10^5^	0.05	0.02
0.4	0.9	10^7^	0.83	0.12
0.4	0.99	10^3^	2.42 × 10^−5^	9.44 × 10^−8^
0.4	0.99	10^5^	0.51	0.21
0.4	0.99	10^7^	0.95	0.04
0.4	0.999	10^3^	0.05	0.02
0.4	0.999	10^5^	0.83	0.12
0.4	0.999	10^7^	0.98	0.01

### Distribution of number of independent significant single-nucleotide polymorphisms

The number of independent significant SNPs is a function of statistical power across all causal SNPs. Testing the significance of each independent SNP could be regarded as a Bernoulli trial $X_{j}$ , which is either 0 or 1, with probability of success rate $s_{j}=\pi_{0}\alpha +\left(1-\pi_{0}\right)\;p_{j}$ , j=1,2,…m, where α is the Type 1 error rate and $p_{j}$ is the statistical power of detecting SNP j . Hence, the total number of significant SNPs $S=\sum_{j=1}^{m}X_{j}$ and its expectation is $E(S)=m\pi_{0}\alpha +\left(1-\pi_{0}\right)E\left(\sum_{j=1}^{m}p_{j}\right)=m[\pi_{0}\alpha +\left(1-\pi_{0}\right)$ E(p)] , where E(p) is the average power of causal SNPs. The expected number of detected causal SNPs $E(C)=\left(1-\pi_{0}\right)E\left(\sum_{j=1}^{m}p_{j}\right)=m\left(1-\pi_{0}\right)$ E(p) *.*

When calculating the variance of the number of significant SNPs, null and non-null SNPs are also considered separately. For null SNPs, the number of significant SNPs is binomial with mean $m\pi_{0}\alpha$ and variance $m\pi_{0}\alpha \left(1-\alpha \right)$ *.* As α is often small in GWAS, the variance is approximately $m\pi_{0}\alpha$ thus the distribution is approximately a Poisson. For non-null SNPs, the number of significant SNPs is a convolution of $m\left(1-\pi_{0}\right)$ Bernoulli trials with different success rates $p_{j}$ , i.e., a Poisson binomial distribution. The variance of the number of significant SNPs is therefore $m\left(1-\pi_{0}\right)\left[E\left(p\right)\left(1-E\left(p\right)\right)-Var\left(p\right)\right]$ , where Var(p) is the variance of power across causal SNPs. Hence, $Var\left(S\right)=m\pi_{0}\alpha \left(1-\alpha \right)+m\left(1-\pi_{0}\right)\left[E\left(p\right)\left(1-E\left(p\right)\right)-Var\left(p\right)\right]$ . This variance is used to construct the 95% probability interval of the number of significant SNPs.

In our model, sample size and $\pi_{0}$ of phenotype are two factors that would affect the number of independent significant SNPs. Specifically, the more polygenic a phenotype is, the smaller the averaged effect size. With the increase of sample size, the smaller the averaged effect size, the slower the expected number of significant SNPs curve plateaus out (Figure 2C).

### Distribution of variance explained by independent significant single-nucleotide polymorphisms

The phenotypic variance explained by independent significant SNPs in a GWAS is $\frac{Var\left(\underset{j\in \Omega }{\sum }\beta_{j}x_{ij}\right)}{Var\left(y_{i}\right)}=\underset{j\in \Omega }{\sum }\beta_{j}^{2},\;i=1,2,\ldots n$ , where Ω denotes the set of such SNPs. However, since the true effect size is unknown, an approximation of the variance explained is $\underset{j\in \Omega }{\sum }{\hat{\beta }}_{j}^{2}$ . This is referred as the apparent variance explained, because substituting β by $\hat{\beta }$ would inflate the result due to Winner’s curse (Palmer and Pe'er, 2017). To correct this overestimation, we use $E\left(\beta_{j}^{2}|{\hat{\beta }}_{j}\right)$ , the possibly best estimator of $\beta_{j}^{2}$ , to replace ${\hat{\beta }}_{j}^{2}$ , i.e., $\underset{j\in \Omega }{\sum }{\left[E\left(\beta_{j}^{2}|{\hat{\beta }}_{j}\right)\right]}^{2}$ .This is referred as the corrected variance explained.

When effect size estimates are calculated in different samples, the number of significant SNPs and ${\hat{\beta }}_{j}$ would vary due to sampling error. In other words, both the number of significant SNPs S and ${\hat{\beta }}_{j}$ are random variables. The expected variance explained by the significant SNPs is

$$E\left(\underset{j\in \Omega }{\sum }{\hat{\beta }}_{j}^{2}\right)=E\left(\sum_{j=1}^{m}X_{j}\right)E\left({\hat{\beta }}_{j}^{2}|{\hat{\beta }}_{j}>T\right)$$

*T* is the critical value given the significance level.

The variance of variance explained by the significant SNPs is obtained using the law of total variance.

$$Var\left(\underset{j\in \Omega }{\sum }{\hat{\beta }}_{j}^{2}\right)=E\left(Var\left(\underset{j\in \Omega }{\sum }{\hat{\beta }}_{j}^{2}|S\right)\right)+Var\left(E\left(\underset{j\in \Omega }{\sum }{\hat{\beta }}_{j}^{2}|S\right)\right)$$

Similarly, the variance of corrected variance explained by significant SNPs can also be calculated.

The relationship between the expected apparent variance explained and sample size shows consistent pattern with that of expected number of significant SNPs and sample size (Figure 2D).

### Simulation results

**FIGURE 3:**
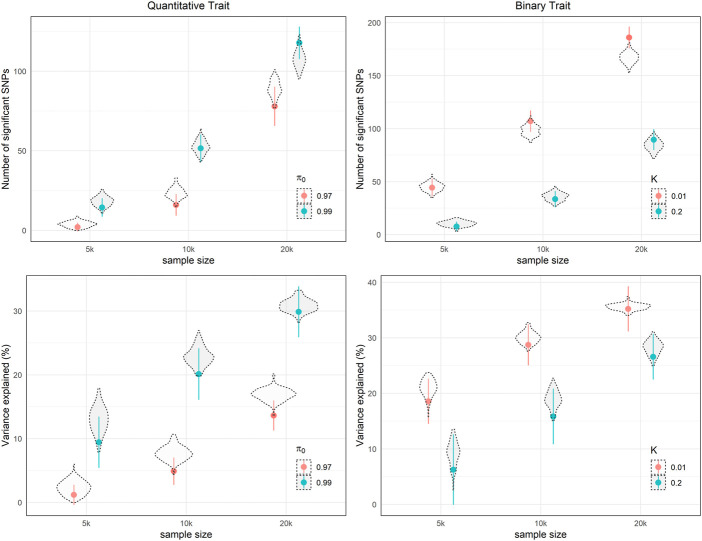
Theoretical expected number of independent significant SNPs and variance explained with 95% probability intervals i.e., dots and whiskers, with different parameters settings in 100 simulations. $h^{2}$ = 0.4, *m* = 50,000, α = 10^–6^. For binary trait, $\pi_{0}$ = 0.99, w = 0.5.

To validate the derived formula, we performed simulation studies using specific genetic architecture parameters (Figure 3). For both continuous and binary phenotypes, the 95% probability intervals of the theoretical number of significant SNPs and variance explained covers the mean of 100-time simulation results, which supports our analytic derivation. In addition, In Table 3, we listed necessary sample sizes to detect 5%, 50%, and 95% of causal SNPs for traits with different levels of $\pi_{0}$ and SNP heritability. It shows that we need disproportional increase of sample size to detect more significant SNPs.

**TABLE 3:** The sample sizes needed to detect 5%, 50%, and 95% of independent significant SNPs for phenotypes with different levels of polygenicity, assuming the effect size following point-normal distribution, *m* = 60,000. *m*_*1*_ is the total number of causal SNPs.

*h* ^*2*^	*π*_*0*_	Total number of independent significant SNPs (*m*_*1*_)	Sample size needed to detect 5% of *m*_*1*_	Sample size needed to detect 50% of *m*_*1*_	Sample size needed to detect 95% of *m*_*1*_
0.1	0.95	3,000	2.02 × 10^5^	1.93 × 10^6^	2.27 × 10^8^
	0.98	1,200	8.08 × 10^4^	7.72 × 10^5^	9.07 × 10^7^
	0.99	600	4.04 × 10^4^	3.86 × 10^5^	4.53 × 10^7^
0.3	0.95	3,000	6.74 × 10^4^	6.43 × 10^5^	7.56 × 10^7^
	0.98	1,200	2.69 × 10^4^	2.57 × 10^5^	3.02 × 10^7^
	0.99	600	1.35 × 10^4^	1.29 × 10^5^	1.51 × 10^7^
0.5	0.95	3,000	4.04 × 10^4^	3.86 × 10^5^	4.53 × 10^7^
	0.98	1,200	1.62 × 10^4^	1.54 × 10^5^	1.81 × 10^7^
	0.99	600	8.08 × 10^3^	7.72 × 10^5^	9.07 × 10^6^

### Application to other study designs

For study design with phenotypic selection of continuous traits, we first consider the extreme phenotype (EP) study design (Barnett et al., 2013), which recruits subjects with extreme phenotypic values from both tail regions of truncated normal distribution ( $Y_{S}$ ). This sampling strategy is shown to be effective for detecting rare variants that contribute to complex traits (Amanat et al., 2020). This is because rare variants are assumed to be enriched in individuals with extreme phenotypic values, and the statistical power to detect these variants is thus increased.

The relationship between sample regression coefficient ${{\hat{\beta }}_{j}}_{S}$ and regression coefficient without phenotypic value selection is ${\hat{\beta }}_{j}=\frac{{{\hat{\beta }}_{j}}_{S}}{var\left(Y_{S}\right)}$ . Under this study design, the equivalent sample size $n^{*}={nVar\left(Y_{S}\right)}^{2}$ , where $Var\left(Y_{S}\right)$ can be calculated by the law of total variance:

$$Var\left(Y_{S}\right)=Var\left(Y|A_{1}\right)P\left(A_{1}\right)+Var\left(Y|A_{2}\right)P\left(A_{2}\right)+E{\left(Y|A_{1}\right)}^{2}\left(1-P\left(A_{1}\right)\right)P\left(A_{1}\right)+E{\left(Y|A_{2}\right)}^{2}\left(1-P\left(A_{2}\right)\right)P\left(A_{2}\right)-2E\left(Y|A_{1}\right)\left(Y|A_{2}\right)P\left(A_{1}\right)P\left(A_{2}\right).$$

$P\left(A_{1}\right)$ is the proportion of samples with extreme small phenotypic values whereas $P\left(A_{2}\right)$ is the proportion of extreme large samples. In fact, this method applies to any method of selection based on Y , not just the truncated normal selection.

Similarly, to calculate the equivalent sample size for case-control study, the key is to build up the relationship between the estimated log odds ratio based on standardised genotype, i.e., γ , and the per-standard deviation effect on the liability scale. The equivalent sample size for a case-control study is $\frac{K^{2}{\left(1-K\right)}^{2}}{w\left(1-w\right)}\frac{1}{{\phi \left(\Phi^{-1}\left(K\right)\right)}^{2}}$ n as mentioned in the Material and methods section.

### Efficacy of polygenic scores is improved using shrinkage method

**FIGURE 4:**
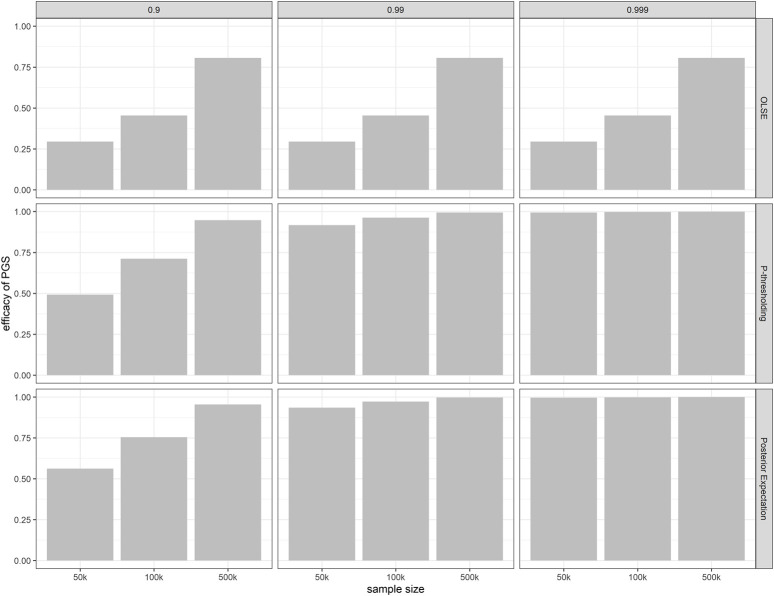
Efficacy of PGS constructed under different $\pi_{0}$ by different methods relative to the true additive genetic value, against sample size. OLSE: ordinary least square estimate. *p*-value threshold is chosen to maximize r^2^. *m* = 60,000. *h* ^*2*^ = 0.5.

Under the assumption of point-normal genetic effect distribution, we also compared the efficacy of PGS constructed by the ordinary least square estimate (OLSE), *p*-value thresholding method and the aforementioned posterior expectation shrinkage relative to the true additive genetic value (Figure 4). In this figure, the *p*-value threshold is chosen to maximize the $r^{2}\left(\hat{G},G\right)$ . When PGS is constructed by OLSE, $\pi_{0}$ would not affect the PGS efficacy. When sample size is large enough, PGS constructed by *p*-value thresholding method can provide efficacious polygenic prediction. However, when the proportion of causal SNPs is high and effect sizes are small, shrinkage method can greatly improve polygenic score efficacy.

### Real data results

We compared the predicted results with the reported meta-GWAS outcomes (Table 4). The predicted number of independent significant SNPs, the apparent and corrected variance explained are calculated based on $\pi_{0}$ such that 95% probability interval of the predicted number of significant SNPs would cover the number reported in earlier GWAS.

**TABLE 4:** Predicted versus reported numbers of independent significant SNPs and variance explained by these SNPs with 95% probability intervals (PIs) based on the range of estimated *π*_*0*_ for height, body mass index (BMI), major depressive disorder (MDD), and schizophrenia (SCZ).

Phenotype (SNP heritability^a^)	Estimated *π*_*0*_ ^b^	Sample size	Number of significant SNPs		Variance explained by significant SNPs (%)		
			Predicted	Reported	Apparent	Corrected	Reported
Height (0.483)	0.66 [0.65, 0.68]	693,529	3466.91 [3380.98, 3547.29]	2388	30.67 [29.24, 32.14]	27.27 [25.87, 28.72]	24.6^d^
BMI (0.249)	0.36 [0.30, 0.40]	681,275	523.47 [419.5, 637.36]	656	3.27 [2.58, 4.05]	2.17 [1.66, 2.76]	6.0^d^
MDD (0.089)	0.85 [0.83, 0.88] (K = 0.15)	305,431^c^	62.2 [31.74,101.84]	44	0.76 [0.37, 1.28]	0.45 [0.19, 0.8]	0.51^e^
	0.88 [0.86, 0.90] (K = 0.25)	262,344^c^	60.34 [31.07, 97.97]		0.86 [0.43, 1.45]	0.53 [0.23, 0.93]	
SCZ (0.23)	0.86 [0.85, 0.87]	265,238^c^	494.71 [430.06, 556.73]	294	8.26 [6.98, 9.55]	6.57 [5.3, 7.55]	2.6


a SNP heritability is on the liability scale for MDD and SCZ.

b $\pi_{0}$ was estimated based on earlier GWAS. Details of calculations are listed in Supplementary Table S3.

c Equivalent sample sizes. Details of calculations are listed in Supplementary Tables S1, S2.

d The reported variance explained included nearly independent SNPs detected using GCTA-COJO, i.e., 3,290 and 941 nearly independent SNPs.

e This value is the average of liability variance explained by SNPs with *p*-value less than 5 × 10^−8^ in row 29, Supplementary Table S4 of Wray et al. (2018).

For BMI and MDD, the predicted key GWAS outcomes are close to the reported values. However, our model over-estimated the results for height and SCZ. For height, one of the possible reasons is that the effect size distribution is not as simple as a point-normal, which is supported by other reference (Zhang et al., 2018). For schizophrenia, mixed population in discovery samples, for example, Asian samples are included in Ripke et al. (2014) and PGC3—SCZ (The Schizophrenia Working Group of the Psychiatric Genomics Consortium Ripke et al., 2020), may lead to the phenomenon that the reported number of significant SNPs is less than expected and it is out of the scope of our model. For different populations, *m* would be different, but how exactly the mixed population in discovery sample would affect the detected number of significant SNPs needs further study.

## Discussion

In this paper, we derived theoretical results and provided computational algorithms for predicting the key outcomes of GWAS or meta-GWAS using parameters regarding the genetic architecture of phenotype and sample size, under the assumption that the standardised effect sizes of all SNPs in the genome follow a point-normal distribution. We conducted simulation studies to validate our theoretical results, and applied our model to GWAS data on four example complex traits.

Our results show that the density function of statistical power across causal SNPs under the assumed effect size distribution is bimodal with peaks near 0 and 1 (a variation of Figure 2B; Supplementary Figure S1). In other words, most causal SNPs have statistical power close to either zero or one, because of “floor” and “ceiling” effects. The relative heights of the two peaks are influenced by sample size; increasing sample size will increase the statistical power of all causal SNPs and thus reduce the height of the peak near zero and increase the height near one. From the distribution of statistical power, the expectations and variances of key GWAS outcomes, such as the number of independent genome-wide significant SNPs and the phenotypic variance explained by these SNPs, can be calculated. These calculations have been implemented in an online interactive tool named Polygenic Power Calculator.

For many phenotypes, meta-GWAS sample sizes have not reached the halfway point of the desired level to detect most of the contributing SNPs. Taking MDD as an example, we estimate that 7.36 × 10 (de Vlaming et al., 2017) equivalent total samples are needed to detect 95% of all causal SNPs when MDD prevalence is 15% whereas the existing equivalent sample size only reaches 3.05 × 10 (Torkamani et al., 2018). On the other hand, it takes a much smaller sample size to capture most of the genetic variance. Figures 2C,D shows that when $\pi_{0}$ is 0.9, i.e., there are 6,000 causal SNPs, it takes ∼10 million samples to detect ∼80% causal SNPs but only takes ∼400 thousand samples to capture ∼80% of SNP heritability. This is because under the assumed normal distribution of causal effects, detecting the SNPs with very small effects requires a very large sample size but does not add very much to variance explained. In practice, with the increase of global collaboration in studying genetics of complex traits, meta-GWAS sample sizes for many phenotypes are steadily increasing. As a result, we would expect to be increasingly able to identify more trait-associated SNPs with small effect sizes. However, we will eventually see a diminishing marginal return in terms of the variance explained and polygenic score prediction accuracy.

In genetic association studies, the most common definition of effect size is the per-allele effect b , estimated by regressing phenotypic value on allele count. However, we adopted the per-standard deviation effect $\beta =\sqrt{2f\left(1-f\right)}b$ , where *f* is the allele frequency. Our assumption that the distribution of *β* is independent of allele frequency implies that per-allele effect sizes are inversely related to SNP variance. Although the per-allele effect has more explicit biological meaning, adopting per-standard deviation effect and assuming this to be independent of allele frequency simplifies power calculation. Indeed, theoretical models and analytical methods of complex trait genetics have widely adopted standardised effect sizes (Yang et al., 2010; Bulik-Sullivan et al., 2015; Privé et al., 2020). It is possible to relax the assumption of independence between standardized effect size and allele frequency; this would then require the allele frequency distribution in the population to be specified. Since the relationship between effect size and allele frequency depends on selective pressure on the phenotype, it is expected to be different for different phenotypes.

The parameter π _0_ in this paper is not equivalent to polygenicity in the usual sense, which usually refers to the proportion of all SNPs that directly influence the phenotypes, and can be estimated by tools such as GENESIS (Zhang et al., 2018) and MiXeR (Holland et al., 2020). Instead, our model makes the simplification of considering only independent SNPs (obtained via linkage disequilibrium pruning or clumping), so that $1-\pi_{0}$ is the proportion of causal SNPs in ∼60,000 nearly independent SNPs. Taking the total number of SNPs in the genome to be approximately 4.5 million (Genomes Project Consortium Auton et al., 2015), each independent SNP on average represents approximately 75 SNPs in the genome. We have assumed that the testing of an equivalent number of independent SNP will have similar properties to the testing of all genotyped and imputable SNPs in current GWAS.

In the early days of GWAS, only a few independent significant SNPs were observed from GWAS and meta-GWAS due to limited sample size. Visscher et al. (Visscher et al., 2012) made the empirical observation of a roughly linear relationship between discovery sample size and the number of genome-wide significant hits, once the sample size reached a level sufficient to detect a few SNPs. This pattern matches the linear part of the S-shape in Figure 2C. In this study, we further extended the range of sample size to that needed to detect nearly all $m\pi_{0}$ independent SNPs, and obtained the predicted relationship in the entire range.

Our method has some limitations. First, we assumed the SNPs to be independent, on the basis that GWAS or meta-GWAS usually report independent SNPs after pruning or clumping. This assumption simplifies the model and bridges the relationship between genetic architecture parameters and key GWAS outcomes directly in a concise manner. We adopted 60,000 as the number of independent SNPs, but the appropriate number may depend on the population, minor allele frequency cutoff, and sample size. A more satisfactory approach in the future may be to explicitly take LD into account, expressing marginal SNP effects by weighted sums of joint effects, while making reasonable assumptions for the joint effect size distribution. Second, we adopted the per standard deviation allele effect as effect size and ignored possible differences in the relationships between allele frequency to effect size distribution for different phenotypes. Although this definition has been widely adopted (Daetwyler et al., 2008; Dudbridge, 2013), models taking allele frequency into account in effect size distribution are not uncommon (Park et al., 2010; So et al., 2010). Third, we assumed the standardised effect sizes followed a point-normal distribution but several other effect size distributions have been proposed (Zhou et al., 2013). Thus, it would be interesting to investigate how these other distributions would alter the predicted behaviour of GWAS outcomes. Fourth, our model ignores the contribution of rare variants (allele frequency < 1%). As GWAS are increasing in both sample size and number of genotyped or imputed SNPs, more rare variants with large effect size are being detected. The observed discrepancies between the predicted values from our model and the reported empirical results for height and schizophrenia also suggest possible inadequacies in our model, including misspecification of effect size distribution, inaccurate estimates of parameters such as π _0_ and *m*, the ignoring of rare variants, and the failure to account for cross-study phenotypic or population heterogeneity in the meta-GWAS.

## Web resources

Heritability of BMI can be found here: http://www.nealelab.is/uk-biobank/. The online power calculator is available at https://twexperiment.shinyapps.io/PPC_v2_1/.

## Data Availability

The original contributions presented in the study are included in the article and its Supplementary Material, further inquiries can be directed to the corresponding author.
