# Supplementary figures and images for: Polygenic power calculator: Statistical power and polygenic prediction accuracy of genome-wide association studies of complex traits
Source: Front Genet. 2022 Oct 10;13:989639. doi: 10.3389/fgene.2022.989639 (PMC9589038; doi:10.3389/fgene.2022.989639)

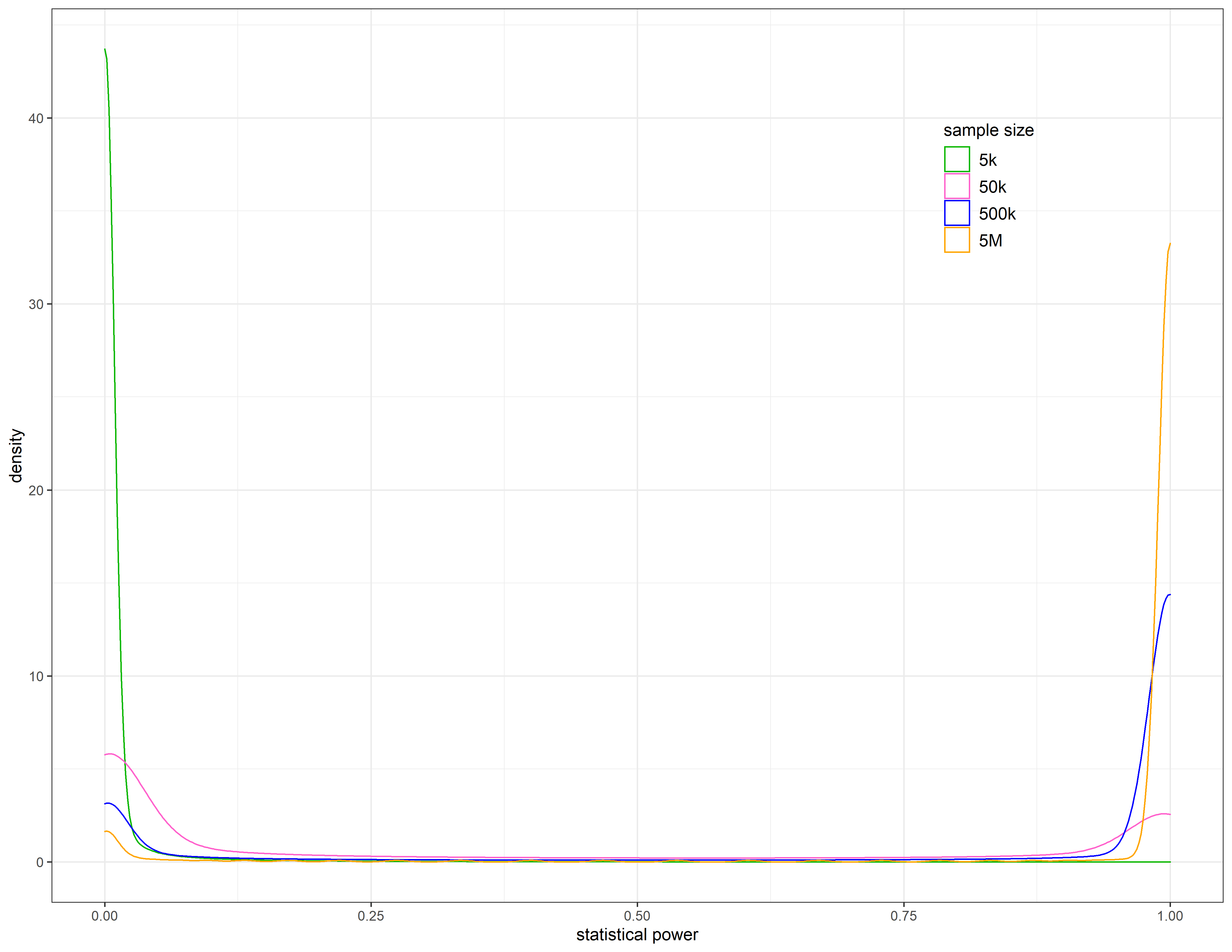

Supplement: Supplementary file 1 [file Image1.TIFF]
